# A Preliminary Study on the Effect of the Instant Controlled Pressure Drop Technology (DIC) on Drying and Rehydration Kinetics of Maize Kernels (*Zea mays* L.)

**DOI:** 10.3390/foods11142151

**Published:** 2022-07-20

**Authors:** Anaberta Cardador-Martínez, Juan Leopoldo Pech-Almeida, Karim Allaf, Natalia Palacios-Rojas, Maritza Alonzo-Macías, Carmen Téllez-Pérez

**Affiliations:** 1 Tecnologico de Monterrey, Escuela de Ingeniería y Ciencias, Epigmenio González 500, Querétaro 76130, Mexico; mcardador@tec.mx (A.C.-M.); juanlpecha@gmail.com (J.L.P.-A.); 2 Laboratory of Engineering Science for Environment LaSIE-UMR-CNRS 7356, Eco-Intensification of Agro-Industrial Eco-Processes, La Rochelle University, 17042 La Rochelle, France; kallaf@univ-lr.fr; 3 International Center for Maize and Wheat Improvement, Carretera México-Veracruz, Texcoco 56237, Mexico; n.palacios@cgiar.org

**Keywords:** Instant Controlled Pressure Drop technology (DIC), maize kernel, drying kinetics, rehydration kinetics, water holding capacity

## Abstract

Maize is one of the three worldwide cereal crops with the most outstanding production; however, its postharvest losses range from 2 to 40% due to inadequate harvesting, drying, and storage technologies. This study focuses on the Instant Controlled Pressure Drop technology (DIC) effect on maize kernels’ drying and rehydration kinetics. In total, 19 different DIC treatments were carried out on maize kernels (~25% d.b.). The DIC parameters studied were steam pressure (0.1 to 0.4 MPa) and treatment time (10 to 90 s). After DIC treatment, drying kinetics were carried out by Convective Air Drying (CAD) at 50 °C and 0.4 ms^−1^ airflow. Rehydration kinetics and Water Holding Capacity (WHC) were evaluated at 20 °C. In comparison to CAD samples, DIC (0.4 MPa and 90 s) reduced the drying time from 180 min to ~108 min. Additionally, regarding the rehydration and WHC results, DIC achieved the same moisture content in only 3.5 min that controls achieved after 1 h of rehydration (0.40 g H_2_O/g dry matter). Moreover, DIC (0.4 MPa and nine cycles of 10 s) increased the WHC 2.3 times compared to the control. In this way, DIC could be a postharvest technology to improve maize kernels’ drying operations and functional properties.

## 1. Introduction

Maize (*Zea mays* L.) is the primary source of calories in the diets of 230 million inhabitants of developing countries, especially in Latin American and African countries [1]. It is one of the three worldwide cereal crops with the most significant production. It has a high value and economic importance worldwide as human food, animal feed, and feedstock for many industrial products and biofuels. As human food, maize is consumed worldwide in various whole and processed products such as pop maize, polenta, tortillas, mush, breakfast cereals, snack food, bakery items, and maize meal [2]. Globally, the USA, China, Brazil, Argentina, Ukraine, Indonesia, India, and Mexico produce three-quarters of the global maize production (881 million tons) [3]. In 2021, Mexico reported an average production of 27.5 million tons and is one of the ten significant consumers of maize with a per capita consumption of 34 kg/year [2,4].

However, low- and middle-income countries’ inadequate postharvest drying and storage operation generates maize losses from 2 to 40% [5]. The major causes of loss are the time of harvest [6], the cracks generated by an inadequate drying operation [7], the changes in functional properties [8,9], and microbial contamination during their development, harvest, or postharvest, especially via fungal attack which triggers the production of mycotoxins in most of the cases [10,11]. Generally, maize is stored for different periods before its utilization after harvesting. Therefore, to extend its shelf life, the initial moisture content of mature maize kernels is reduced from around 54% dry basis (d.b.) to 15% d.b. Shelled maize can be stored safely for a short time at 17% d.b.; however, for storage of 6–12 months, the maximum safe moisture content is 15% d.b. Then, for long-term storage (more than a year), it should be dried to below 15% d.b. As a living entity composed mostly of carbohydrates and a variety of microflora, when maize moisture is between 54 and 20% d.b., conditions are favorable for the rapid growth of storage fungi that can damage or destroy maize kernels [2].

Before World War II, most maize raised for grain was harvested on the ear and preserved by naturally air-drying in slotted cribs on farms, taking up to 100 days for the kernels to dry to storage-safe humidity contents. However, because cribs were kept open to allow air circulation, they also admitted rain, birds, rodents, and insects, and these intrusions resulted in losses in maize quality and quantity. Then, as maize producers were faced with storing masses of shelled maize at moistures too high for safe storage, the artificial drying of maize became the most common practice to lower the moisture content of the grain [2].

Although artificial drying has been economically feasible since 1945, postharvest losses of up to 40% in developing countries and 9% in developed countries have been reported in this decade. Postharvest maize losses range from 2 to 40% in low and middle-income countries due to a lack of adequate harvesting, drying, and storage technologies and a lack of education on good practices [12]. The most crucial kernel deterioration factors are the heterogeneity of maize moisture content in bins during storage, the stress cracks of kernels, mycotoxin occurrence, and the invasion of insects, birds, and rodents [2].

Regardless of the dehydration technique, except freeze-drying, the principal studied factors to optimize maize drying have been air temperature [13], relative humidity [14], and airflow velocity [12]. However, the kernels’ fundamental aspects of heat and mass transfers have not been well evaluated. Traditional drying methods of cereals are characterized by low kinetics, an inevitable shrinkage of the product structure, and nutritional losses due to the long drying periods. Moreover, these techniques do not eliminate microbial contamination, much less remove fungal spores.

The main purposes of the maize drying operation are: (1) to reduce the moisture content according to the commercialization standard; (2) to minimize chemical and microbiological activity to promote storage; (3) to preserve the nutritional, biological, and end-use properties of the product; (4) to extend the useful life of the product; (5) to minimize losses of dry matter; and (6) to reduce the mass to facilitate transport. Therefore, an inefficient drying operation can affect the quality of the maize from the physical, biological, chemical, and organoleptic points of view and generate high economic losses [2].

The first thing that happens during a drying process is a transfer of heat from the air to the grain, which is mostly used to evaporate the moisture from the grain surface. Once the water has evaporated on the surface, the diffusion process begins, consisting of the movement of water from the inside of the grain towards the surface through diffusion. The diffusion process is slower than the evaporation process. There is always a moisture gradient inside the grain; the maximum humidity is in the center, while the minimum is on the surface. Therefore, the limiting factor of the drying process is the diffusivity of the water [15].

Over many years, hundreds of studies have focused on optimizing the drying process by increasing air temperature and velocity and coupling new energy sources to accelerate heat transfer [7,16,17,18]. However, none of them has considered the internal mass transfer diffusion as the limiting factor. In this respect, the Instant Controlled Pressure Drop technology, well known by its French acronym DIC (Détente Instantanée Contrôlée), has allowed for controlling the porosity of various biological products such as fruit, vegetables, and medicinal plants leading to higher diffusivity, a much lower drying time, and significantly faster rehydration kinetics [19,20,21].

The DIC treatment is a five-step thermo-mechanical treatment based on a short-time heat treatment (0.1–0.9 MPa for some seconds) followed by an abrupt pressure drop, at a ratio of over 0.5 MPa per second, leading to a final absolute pressure of 10 to 5 kPa [22], compared to 101.325 kPa which is the atmospheric pressure at sea level. The controlled abrupt pressure drop is the core of DIC technology, as it triggers water auto-vaporization, instant cooling of biological products, and creates alveoli in the matrix [22].

Therefore, this study’s objective was to conduct preliminary research on the coupling of the Instant Controlled Pressure Drop technology (DIC) to traditional Convective Air Drying (CAD) to evaluate their impact on the drying and rehydration kinetics of maize.

## 2. Materials and Methods

### 2.1. Materials

This study used a commertial hybrid maize, recognized as a white dented grain destined for animal feed with an excellent cob uniformity [23]. Maize was obtained from the International Maize and Wheat Improvement Center (CIMMYT (for its initials in Spanish), and it was collected from experimental trials at the state of Tamaulipas, Mexico (Abasolo 24°03′21″ N-98°22′24″ W). For this study, maize was planted on 11 February 2020 and harvested on 30 June 2020. The kernels’ harvested moisture content was 12.9% d.b. and the maize was stored in the laboratory at 5 °C before any treatment occurred.

### 2.2. Methods

#### 2.2.1. Sample Preparation

Before drying treatments, good-quality grains (absent of mold and insect contamination) were manually selected. Then, to study dehydration kinetics, kernels with an initial moisture content of 12.9% d.b. were rehydrated to 25% d.b. To ensure moisture content homogeneity, samples were stored for two days at 5 °C. Subsequently, rehydrated maize kernels were divided into twenty-two lots: three for Convective Air Drying (CAD), which were used as a control, and nineteen for DIC coupled to CAD.

#### 2.2.2. Drying Methods

##### Convective Air Drying (CAD)

Convective air drying of 200 g of maize kernels at 25% d.b. was applied in a cabinet dryer (SEM-1 model, POLINOX, Mexico) at 50 °C, 265 Pa initial partial pressure of vapor in the air flux, and air velocity of 0.4 ms^−1^ for 24 h. The drying process was carried out in triplicate and the obtained samples were used as controls.

##### Instant Controlled Pressure Drop (DIC) Coupled with Convective Air Drying (CAD)

**Figure 1:**
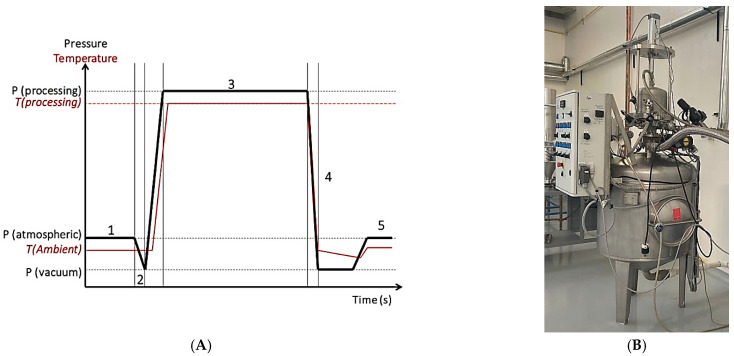
(**A**) Schematic of the time-temperature-pressure profile of a DIC processing cycle. (1) Establishment of the vacuum within the processing reactor; (2) injection of steam at the selected pressure; (3) maintenance of treatment pressure during selected time; (4) instant controlled pressure drop towards vacuum; and (5) establishment of the atmospheric pressure within the processing reactor. (**B**) Laboratory DIC reactor (LABIC0.1, ABCAR-DIC Process; La Rochelle, France).

For the Instant Controlled Pressure Drop treatment, 200 g of maize kernel at an initial moisture content of 25% w.b. were placed into a DIC reactor under atmospheric pressure (Figure 1 stage 1). Then, as can be observed in Figure 1, the vacuum was established in the reactor until a final absolute pressure of 10 to 5 kPa (Figure 1, stage 2) was obtained. After that, the samples were subjected to hydrothermal processing which consisted of injecting saturated steam into the DIC reactor until the targeted steam pressure and treatment time were achieved (Figure 1, stage 3). It must be stated that the treatment time began only when the saturated steam pressure has been achieved. This study selected steam pressure and treatment times according to the experimental design (Table 1). In the fourth stage, at the heart of the DIC technology, an abrupt pressure drop towards a vacuum (between 10 and 5 kPa) at a rate of 5 MPa/s (Figure 1, stage 4) occurred. The final stage consisted of going back to atmospheric conditions inside the DIC reactor (Figure 1, stage 5). It is worth highlighting that applying more than one DIC cycle could be possible according to the characteristics of biological matrices and the objectives pursued. Then, steps 2 through 4 could be repeated for the required number of cycles. This study used a laboratory-sized DIC reactor (LABIC0.1, ABCAR-DIC Process; La Rochelle, France). Figure 1 shows the schematic time-temperatures-pressures profiles of a DIC processing cycle (A) and the laboratory DIC reactor (B). After DIC treatment, the moisture content of samples was measured, and they were completely dried under the same conditions as CAD samples.

#### 2.2.3. Drying and Rehydration Kinetics

Drying kinetics were carried on 30 ± 0.5 g of maize kernels. During drying, samples were weighed at regular intervals throughout a total drying period of 24 h. The weight of the samples was recorded every 5 min (as interval time) during the first 30 min, then at 45, 60, 90, 120, 150, and 180 min. After that, samples were weighed after 24 of drying to achieve an equilibrium water content. Moreover, using a digital caliper, ten maize kernels’ length, width, and thickness were measured at the end of the drying kinetics. At the end of the operation, the moisture content of all dried samples was measured.

For rehydration kinetics, 3 g of each dried sample were placed into tea strainers and submerged in a vessel containing distilled water at room temperature (25 °C). To study the rehydration kinetics, the tea strainers were first soaked, and after they were strained, blotted with tissue paper to remove superficial water, and weighed. The measurements were done every 5 min for the first 15 min and every 15 min up to 60 min, then measurements were taken every 30 min until 3 h. At the end of the rehydration operation, the final moisture content of the samples was measured.

#### 2.2.4. Mathematical Modeling of Drying and Rehydration Kinetics

The study of Mounir and Allaf [24] was adopted to model the dehydration kinetics of maize. According to this model, four physical transfer mechanisms occur during drying: (1) external heat transfer, (2) internal heat transfer, (3) internal water transfer, and (4) external water transport. Thus, only internal transfers may intervene as limiting processes when external heat and mass transfers do not limit the whole operation, meaning adequate airflow conditions in temperature, moisture content, and airflow velocity [25]. In such conditions, the model proposed by Mounir and Allaf [24] was adopted with a Fick-type relation [26]:

(1) $$\frac{\rho_{w}}{\rho_{m}}\left(\vec{\nu_{w}}-\vec{\nu_{m}}\right)=-D_{eff}\vec{\nabla }\left(\frac{\rho_{w}}{\rho_{m}}\right),$$

Then, by assuming any structure modification (*ρ_m_* = constant and *v_m_* = 0), Equation (1) becomes:

(2) $$\rho_{w}\vec{\upsilon_{w}}=-D_{eff}\vec{\nabla }\rho_{w},$$

Then, by using the balance mass, the second Fick law is obtained:

(3) $$\frac{\partial \rho_{W}}{\partial t}=\vec{\nabla }\cdot D_{eff}\vec{\nabla }\rho_{w},$$

By assuming the hypothesis of both structural and thermal homogeneities:

(4) $$\frac{\partial \rho_{W}}{\partial t}=D_{eff}\vec{\nabla }\cdot \vec{\nabla }\rho_{w},$$

Including a one-dimensional flow, the whole process is controlled by only mass transfer:

(5) $$\frac{\partial \rho_{W}}{\partial t}=D_{eff}\frac{\partial^{2}\rho_{w}}{\partial x^{2}},$$

It has been remarked that the provided solutions to this diffusion equation closely depend on the initial and boundary conditions. Then, in this study, Crank’s solution was adopted [27]:

(6) $$\frac{W_{\infty }-W}{W_{\infty }{-W}_{1}}=\sum_{i=1}^{}A_{i}exp\left(-q_{i}^{2}\tau \right),$$

where *W*, *W_∞_*, and *W*_1_ are the amounts of water content (d.b) in the solid matrix at time *t* (*W*); *W_∞_* water content at equilibrium at very long time *t* → ∞; and *W*_1_ water content at the starting diffusion time.

*W*_1_ is the value of *W* at the time *t*_1_ chosen as the beginning of the diffusion model and is used only for long-time experiments.

(7) $$\frac{W_{\infty }-W}{W_{\infty }-W_{1}}=\overset{\infty }{\underset{1}{\sum }}A_{i}\exp\left(-q_{i}t\right)=\frac{8}{\pi^{2}}exp^{-\frac{\pi^{2}D_{eff}t}{4d_{p}{}^{2}}}+\frac{8}{9\pi^{2}}exp^{-\frac{9\pi^{2}D_{eff}t}{4d_{p}{}^{2}}}+\frac{8}{25\pi^{2}}exp^{-\frac{25\pi^{2}D_{eff}t}{4d_{p}{}^{2}}}+\frac{8}{49\pi^{2}}exp^{-\frac{49\pi^{2}D_{eff}t}{4d_{p}{}^{2}}}+\ldots$$

Coefficients of Crank solutions *A_i_* and *q_i_* are given according to the matrix geometry Fick’s number (*τ*) defined as:

*Τ* = *D_eff_* × *t*/*d*_*p*_^2^, (8)

where *d_p_* is the characteristic length (m). For this case, as suggested by Muthukumarappan and Gunasekaran [28], infinite plate geometry is considered, and *d_p_* is the half-thickness of maize. By limiting Equation (7) to its first term, it could be expressed as:

(9) $$\frac{W_{\infty }-W}{W_{\infty }-W_{0}}=\mathrm{Aexp}(-\mathrm{kt}),$$

The logarithmic representation of Equation (9) as a straight line leads to determining *D_eff_* from the slope *k*:

(10) $$LN\left(Y\right)=LN\left(\frac{W_{\infty }-W}{W_{\infty }-W_{0}}\right)=kt,$$

where *k* corresponds to:

(11) $$k=\frac{\pi^{2}D_{eff}}{4d_{p}{}^{2}},$$

So the effective diffusivity is:

(12) $$D_{eff}=\frac{4d_{p}{}^{2}}{\pi^{2}}k,$$

By taking into account that a quantity of water is removed from the surface independently of diffusion processes during the initial minutes of drying, this model excludes the points close to *t* = 0 to determine the effective diffusivity of the experimental data. On the other hand, to evaluate this quantity of water removed from the surface, the extrapolation of the model allows for determining the *W*_0_, which is generally different from the initial humidity content *W_i_*. Therefore, this difference between *W_i_* and *W*_0_ has been defined as the “starting accessibility of water” and reveals the humidity quickly removed from the surface.

When the diffusion process does not control the drying operation, it cannot be possible to determine the effective diffusivity from the experimental data. Then, in this case, the obtained results from Equation (12) can be considered an apparent drying coefficient (D_app_). The same equations apply to rehydration kinetics.

#### 2.2.5. Experimental Design for DIC Treatment

To reduce experimental points, a central composite rotatable design with two-independent variables (*n* = 2), steam pressure “P” (MPa) and thermal treatment time “t” (s), and five levels (−α, −1, 0, +1, and +α) were used. The design included eight total experiments plus five repetitions of the central points (0,0). The value of α (axial distance), depending on the number of parameters considered (n), was calculated as α = (2n)^0.25^. Then, for this study, α = 1.4142. Finally, to evaluate the effect of the highest values of steam pressure and treatment time, the combination of +α points (+α, +α) was also studied. In this respect, treatment time was evaluated under two configurations: (i) a DIC treatment under +α,+α during only one treatment cycle and (ii) a DIC treatment under +α, +α divided into nine cycles. Table 1 shows all the evaluated experiments.

To analyze the results data, the analysis of variance (ANOVA) was used to determine the significant differences between the independent variables (*p* ≤ 0.05), and Pareto charts were used as a graphical tool to identify the impact of variables on responses. To optimize the response, surface response plots were used, and in the case that the R² of fitting models were accurate enough to real data, empirical models were presented. Statistica 13.0 was used to analyze all data.

#### 2.2.6. Assessment Methods

##### Proximate Analysis of Raw Material

Proximate analysis was performed on only raw material according to AACC methods. Moisture content (Method 44-14a from the AACC), ash content (Method 08-12 from the AACC), crude protein (Kjeldahl method), crude fat (Goldfisch method), and crude fiber (Ankom crude fiber method) [29] were used.

##### Thousand Kernel Weight

For this analysis, the method of Navarro, et al. [14] was adopted. Grain samples were sorted and cleaned to remove foreign material and broken kernels. Then, 100 kernels were manually selected and counted further to weigh the sample with an accuracy of 0.01 g. To obtain the 1000 kernel weight in grams, the obtained results were multiplied by 10.

##### Water Holding Capacity

To measure the Water Holding Capacity (WHC) of maize kernels, 5 g of dried samples were ground for 5 min in a blender (BPCT02-BA0-000, Oster, Philippines). Then, 2.5 g of the ground sample was vigorously vortexed with 22.5 g of cold distilled water for 1 min in a 50 mL conical tube (Falcon). After that, the samples were incubated for one hour at ambient temperature (25 °C). They were then centrifuged for 30 min at 3500 rpm at ambient temperature to eliminate the supernatant. A second centrifugation was done for 5 min, the second supernatant was eliminated, and the weight and the moisture content of the final sample were measured. The water holding capacity was calculated as:

$$WHC=\frac{\;\mathrm{Weight}\;\mathrm{of}\;\mathrm{absorbed}\;\mathrm{water}}{\mathrm{Weight}\;\mathrm{of}\;\mathrm{dry}\;\mathrm{sample}},$$

## 3. Results

### 3.1. Proximate Analysis

Proximate analysis was conducted to examine the chemical compositions of raw material maize kernel grains. The result showed that the analyzed grains presented 11.35 ± 2.58% of moisture content on a wet basis, equivalent to 12.9% on a dry basis, 8.91 ± 1.48% of crude protein, 1.69 ± 0.18% of crude fat, 0.89 ± 0.05% of crude fiber, 0.86 ± 0.05% of ash, and 76.3% (calculated by the difference) of total carbohydrates.

### 3.2. Thousand Kernel Weight

**Figure 2:**
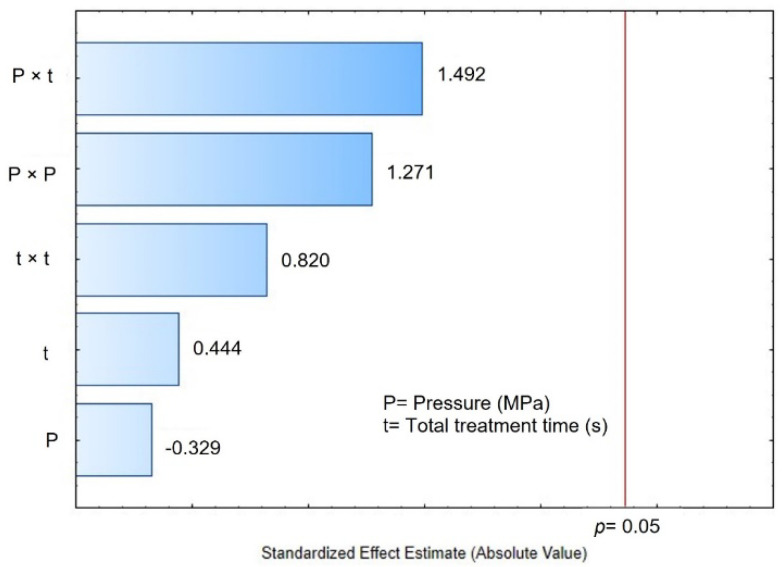
Pareto chart: effects of steam pressure (MPa) and treatment time (s) of DIC treatment on the thousand kernel weight (g) of maize kernels.

To evaluate the effect of DIC treatment on the seed size, the thousand kernel weight in grams is shown in Table 2. Controls presented an average of 272.02 g, and DIC-CAD samples achieved values between 236.45 and 311.77 g. Figure 2 illustrates that selected DIC variables (saturated steam pressure and thermal treatment time) cannot accurately explain this response under the range of the selected parameters.

### 3.3. Drying Kinetics

**Figure 3:**
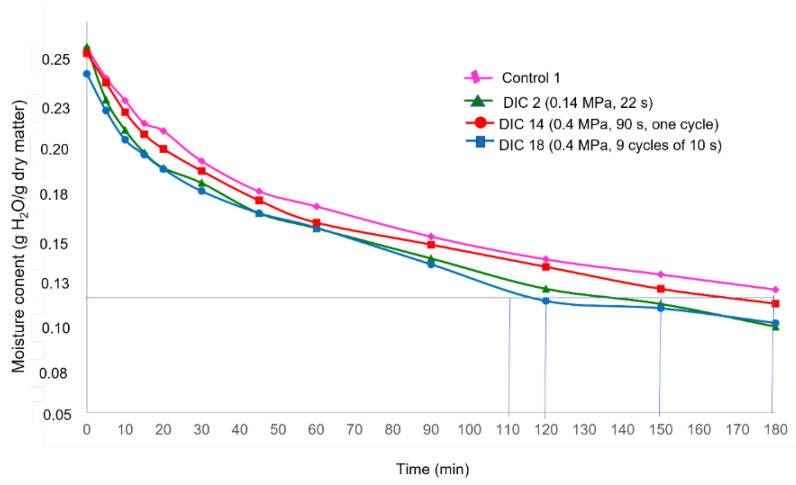
Drying curves of maize kernels W = f(t): Control and DIC-CAD (P = 0.14 MPa and t = 22 s; P = 0.4 MPa and t = 90 s and P = 0.4 MPa and t = 90 s divided into nine cycles of 10 s).

The drying kinetics of maize kernels were studied from an initial average water content of 0.25 ± 0.01 g to approximately 0.10 ± 0.01 g H_2_O/g dry matter. Table 3 shows the evolution of moisture content (d.b) as a function of the time W = f(t) obtained from experimental drying kinetics. Figure 3 shows the drying curves W = f(t) of a control (CAD) and some DIC-CAD maize kernels and illustrates, under specific operating conditions of DIC treatment (DIC 2: 0.14 MPa and 22 s; DIC 14: 0.4 MPa and 90 s with only one cycle of treatment and DIC 18: 0.4 MPa and nine cycles of 10 s), the drying kinetics of maize kernels were improved with respect to the control. In fact, while controls required around 180 min to achieve a final moisture content of 0.12 g H_2_O/g dry matter, DIC 2 required around 120 min, DIC 18 required 150 min, and DIC 14 required around 108 min).

**Figure 4:**
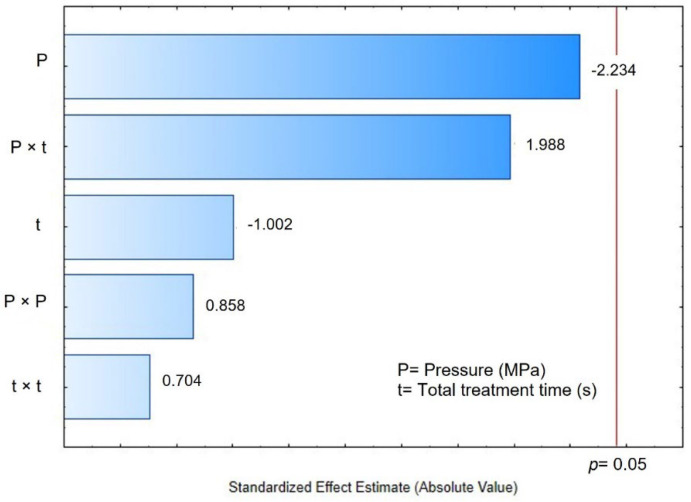
Pareto chart: the effects of steam pressure (MPa) and time (s) of DIC treatment on the apparent drying coefficient (10^−10^ m²/s) of maize kernels.

Moreover, to better evaluate the effect of DIC treatment on the drying kinetics, the apparent drying coefficient (D_app_) was calculated. Table 2 presents the apparent drying coefficient of the controls and DIC-CAD maize kernels. Controls presented an average measure of 0.3364 × 10^−10^ m²/s and DIC-CAD samples obtained values between 0.1738 and 0.3741 × 10^−10^ m²/s. The lowest D_app_ (0.1738 × 10^−10^ m²/s) was achieved under DIC 9 (0.25 MPa and 50 s) and the highest D_app_ (0.3741 × 10^−10^ m²/s) under DIC 14 (0.4 MPa and 90 s only one cycle). These results suggest that DIC could modify the apparent drying coefficient; however, regarding the Pareto chart in Figure 4, it can be concluded that under the selected operating parameters (saturated steam pressure and thermal treatment time), these variables cannot accurately explain this response.

### 3.4. Rehydration Kinetics

**Figure 5:**
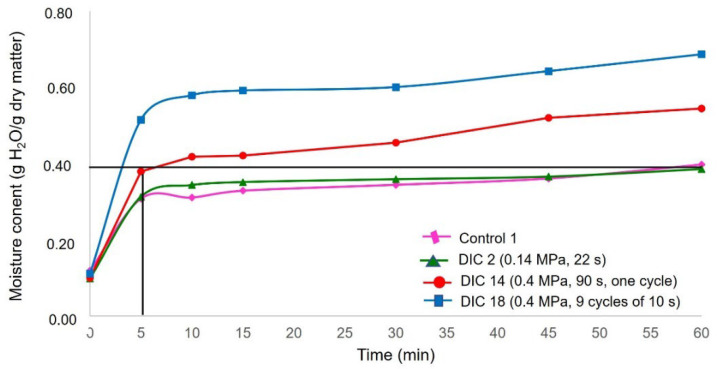
Rehydration curves of maize kernels: Control and DIC-CAD (P = 0.14 MPa, t = 22 s; P = 0.4 MPa, t = 90 s and P = 0.4 MPa, t = 90 s divided into nine cycles of 10 s).

**Figure 6:**
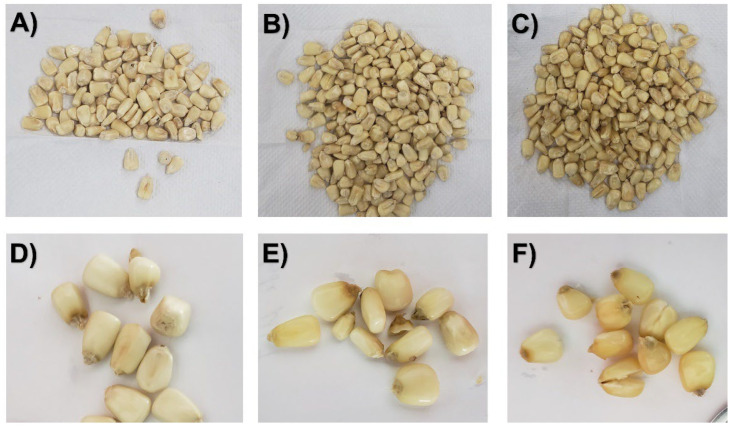
Dried and rehydrated maize kernels: (**A**) Dried control, (**B**) Dried DIC 14 (P = 0.4 MPa, t = 90 s), (**C**) Dried DIC 18 (P = 0.4 MPa, t = 90 s divided into nine cycles of 10 s), (**D**) Rehydrated Control, (**E**) Rehydrated DIC 14, and (**F**) Rehydrated DIC 18.

The rehydration kinetics were studied from an initial water content of maize kernels of 0.10 ± 0.01 g H_2_O/g dry matter for 180 min. Table 4 shows the evolution of moisture content (d.b) as a function of the time W = f(t) obtained from experimental rehydration kinetics. Figure 5 shows the rehydration kinetics of a control (CAD) and some DIC-CAD maize kernels. As can be observed from Table 4, after 180 min of rehydration, controls achieved an average final moisture content of 0.50 g H_2_O/g dry matter, while DIC-CAD samples achieved a final moisture content ranging from 0.43 to 0.84 g H_2_O/g dry matter. The lowest final moisture content was obtained under DIC 3 (0.14 MPa and 78 s) and the highest value by DIC 18 (0.4 MPa and 9 cycles of 10 s). On the other hand, regarding the rehydration curves from Figure 5, the rehydration kinetics of maize were significantly improved with respect to the control under DIC 14 and DIC 18. In fact, while controls required 60 min to achieve a final moisture content of 0.40 g H_2_O/g dry matter, DIC 14 required ~6 min, and DIC 18 required ~3.5 min. Figure 6 shows the control, DIC 14, and DIC 18 of dried and rehydrated maize.

To better evaluate the effect of DIC treatment on the rehydration kinetics of maize kernels, the effective diffusivity (Deff_rehy), the starting accessibility (δW_s,rehy_), and the moisture content after 2 h of rehydration (W_t = 120 min_) were also studied. Table 5 shows the D_eff_rehy_, δW_s, rehy_, and W_t = 120 min_ for controls and DIC-CAD maize kernels

**Figure 7:**
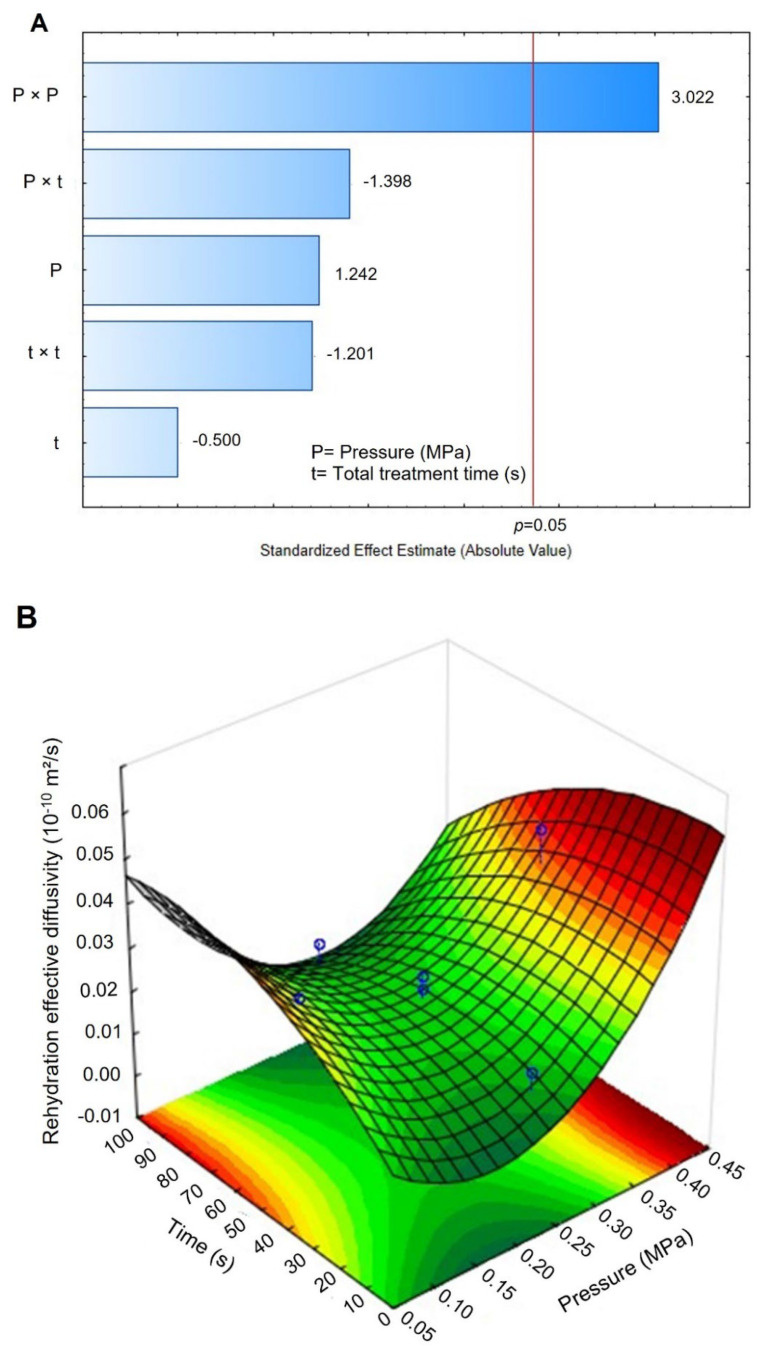
Effects of Pressure (MPa) and time (s) of DIC treatment on the rehydration effective diffusivity (10^−10^ m²/s) in maize kernels. (**A**) Pareto Chart and (**B**) surface response and fitting model. D_eff-rehy_ = 0.0202 − 0.230P + 0.664P^2^ + 0.00071t − 0.0000037t^2^ − 0.001509P × t.

Concerning the effective diffusivity during rehydration (D_eff_rehy_), controls presented an average measure of 0.0146 × 10^−10^ m²/s, and DIC-CAD samples presented values between 0.0030 and 0.0385 × 10^−10^ m²/s. The lowest D_eff_rehy_ was obtained under DIC 7 (0.25 MPa and 50 s) and the highest values under DIC 13 (0.4 MPa and 50 s). Figure 7A shows that under the selected operating parameters (P and t), the saturated steam pressure square significantly affected the D_eff_rehy_ response. Additionally, the response surface graph (Figure 7B) shows that the D_eff_rehy_ could be increased under steam pressure above 0.40 MPa and a treatment time of some seconds. Figure 7B also displays the fitting model for this response.

On the other hand, regarding the starting accessibility response, controls presented an average of 0.2134 g H_2_O/g dry matter and DIC-CAD samples presented values between 0.1695 and 0.3941 g H_2_O/g dry matter (Table 5). The lowest values were achieved by DIC 5 (0.25 MPa and 50 s) and the highest under DIC 17 (0.4 MPa and nine cycles of 10 s). During rehydration kinetics, starting accessibility was defined as the quantity of water that could be added to the product’s surface before water diffusion occurs. In this respect, Table 3 suggests that DIC could modify the δWs; however, regarding the Pareto chart of Figure 4, it can be concluded that under the selected operating parameters (P and t) these variables do not accurately explain this response.

**Figure 8:**
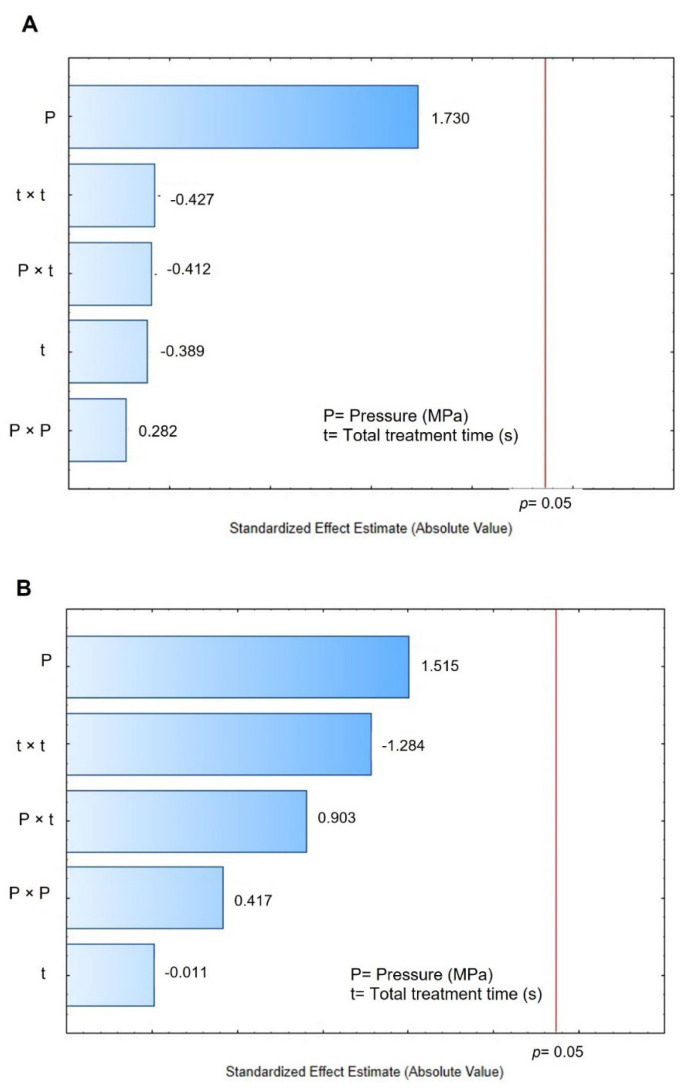
Effects of Pressure (MPa) and time (s) of DIC treatment on (**A**) starting accessibility of maize kernels during rehydration, and (**B**) water content of maize kernels after 2 h of rehydration.

Finally, by comparing the final moisture content after 2 h of rehydration (W_t = 120 min_) of the control (0.442 g H_2_O/g dry matter) vs. DIC-CAD samples (0.3917 to 0.7530 g H_2_O/g dry matter), it can be observed that under DIC treatments of 0.4 MPa and nine cycles of 10 s it could be possible to increase significantly the final moisture content (1.7 times for DIC 17 vs. control). However, regarding the Pareto chart (Figure 8B), the W_t = 120 min_ cannot be accurately explained by steam pressure and treatment time under the selected ranges.

### 3.5. Water Holding Capacity

**Figure 9:**
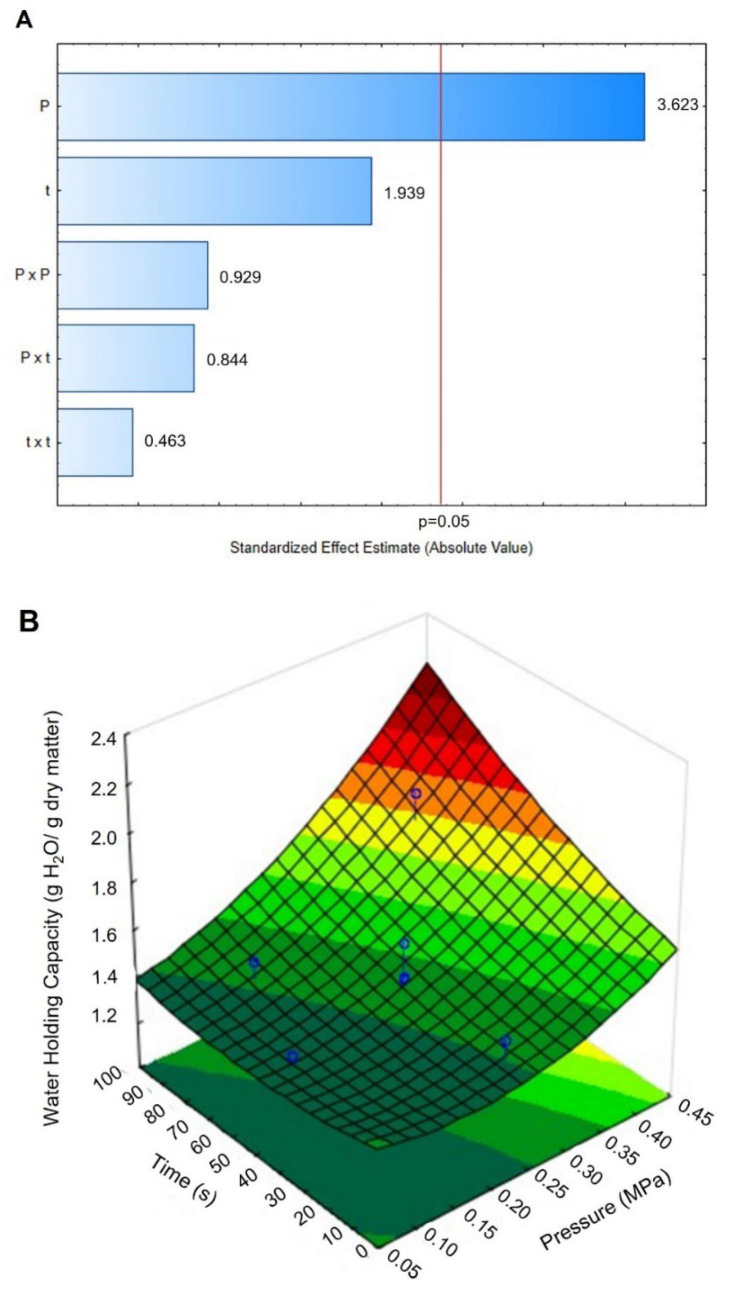
Effects of Pressure (MPa) and time (s) of DIC treatment on the water holding capacity (WHC) of maize kernels. (**A**) Pareto Chart and (**B**) surface response and fitting model. WHC = 1.146 − 1.114P + 3.306P^2^ − 0.0034t + 0.000023t^2^ + 0.0147P × t.

The water holding capacity (WHC) was studied as a quality parameter to evaluate the structural tissue damage caused by the different drying techniques; the higher the WHC, the better the structural preservation. Table 5 shows the WHC results obtained for the control and DIC-CAD maize kernels. Controls obtained WHC values of 1.4304 g H_2_O/g dry matter and DIC-CAD samples obtained values between 1.2859 and 3.5073 g H_2_O/g dry matter. Figure 9A shows that under the selected operating parameters of DIC treatment, the WHC can be accurately explained by the linear effect of the steam pressure. The higher the steam pressure, the higher the WHC. Figure 9B shows the response surface graphic and the fitting model for this effect.

## 4. Discussion

### 4.1. Proximate Analysis

According to various studies, the average maize kernel is made up of about 72% carbohydrate, 9% protein, 5% lipid, and 2% fiber [30,31]. Then, the slight variations between data obtained from this study to previous reports can be linked to the differences within and between maize varieties. Moreover, the variety used in this study belongs to the dent variety, which is characterized by a vitreous endosperm at the sides and back of the kernel and a soft and floury endosperm at the central core extending to the crown [32,33].

### 4.2. Thousand Kernel Weight

According to the results, under selected parameters of DIC treatment, the thousand kernel weight does not change significantly among studied samples, except for DIC 2 (0.14 MPa, 22 s) which presented 311.77 g. This variation could be linked to the final moisture content of this sample at the end of drying. The grain moisture loss is reflected in weight loss. According to Karthik et al. [34], the thousand kernel weight of maize can range from 81 to 322 g.

It is suggested to evaluate the apparent density and the relative expansion ratio of raw materials and DIC-treated samples in a future study. The apparent density, defined as the relationship between the mass and volume of the material (which includes pore volume and water), and the relative expansion ratio, defined as a volumetric ratio between DIC and conventional hot air-dried products [35,36,37,38], will help to better understand the impact of DIC processing parameters (steam pressure and treatment time) on the new microporous structure generated by the auto vaporization process.

### 4.3. Drying Kinetics

The experimental drying kinetic data shows that under the selected conditions of temperature (50 °C) and air flux (0.4 m s^−1^), it cannot be assumed that external heat and mass transfers do not limit the drying operation. As indicated by Nguyen et al. [20], when the air velocity is lower than a critical airflow velocity (CAV), the external transfer limits the drying processes. Thus, it cannot be possible to calculate the effective diffusivity from drying curves W = *f*(t). This statement agrees with the study of Prachayawarakorn et al., 2004 [7], who determined that under an air flux of 3 m/s and temperatures of 90, 110, 130, 150 and 170 °C, it was possible to determine the D_eff_ of maize during fluidized-bed drying, indicating that under these conditions moisture movement inside the maize kernel is controlled by the internal diffusion of water. Moreover, as the starting accessibility reveals a first airflow/interaction surface, a “washing stage” before diffusion controls the drying process, and to calculate it, it is necessary to determine the W_0_ from a diffusional model. Again, however, the starting accessibility could not be calculated. Therefore, this study determined the apparent drying coefficient (D_app_) which involves both washing and diffusion stages. Then, regarding the obtained results of the apparent drying coefficient from CAD and DIC-CAD samples, it can be remarked that under DIC 14 (0.4 MPa and 90 s), the apparent drying coefficient was slightly increased (0.3741 × 10^−10^ m^2^ s^−1^) with respect to the control (0.3364 m^2^ s^−1^). Regarding the drying curves W = f(t) from Figure 3, it can be observed that under DIC 14, the drying time to achieve the same final drying content of controls (0.12 g H_2_O/g dry matter) can be reduced from 180 min to ~105 min. Thus, this preliminary study allowed us to determine that a DIC pretreatment before CAD could be a postharvest technology to reduce the drying time; however, to better determine the effect of DIC treatment, it will be necessary to guarantee negligible external resistances to drying processes. For that, it is necessary to ensure a high airflow temperature and velocity with low relative humidity and adequate interaction surface. Then, it will be necessary to study the drying kinetics of DIC-CAD samples under at least three different temperatures (i.e., 40, 50, and 60 °C) and air flux up to CAV. In this respect, the studies of Akowuah [39] and Owusu-Sekyere [40] determined that the optimal drying conditions to avoid kernel damage and maintain the viability of maize kernels were 50 °C and an air flux of 2.5 m/s. However, a deep study will be necessary to determine the CAV of freshly harvested maize at ~0.25 g H_2_O/g dry.

### 4.4. Rehydration Kinetics

Rehydration is a crucial step prior to various maize kernel processing such as cooking, fermentation, germination, nixtamalization, etc. However, this process used to be long which could cause microbial spoilage, swelling, and physical destruction of the food [41]. According to Martínez-Garza [42], obtaining a complete rehydrated grain sometimes requires 16–36 h, indicating that new technologies are needed to accelerate this process. In this respect, this study showed that the DIC technology could significantly improve the rehydration of maize kernels. As shown in Table 3, the D_eff_rehy_ of maize control kernels was improved 2.6 times by DIC 2 (0.14 MPa, 22 s), from 0.0146 to 0.0385 10^−10^ m²/s. This result could be explained by reducing grain tortuosity triggered by the instant controlled pressure drop. Moreover, the response surface plot from Figure 7B allowed us to identify that under high steam pressure treatments (between 0.37 and 0.45 MPa) and short treatment times (between 10 and 60 s), it could be possible to optimize the rehydration of maize kernels at the diffusional stage. In addition, regarding the starting accessibility, it can also be highlighted that, under DIC treatments at 0.4 MPa and nine cycles of 10 s, it was possible to intensify the quantity of water that could be absorbed on the surface of the grain to 1.8 times higher than that in controls (0.2134 vs. 0.3941 g H_2_O/g dry matter). Similar results have been found in fruits [43], vegetables [19], and meat [44] treated by DIC. These results may be due to the swelling of maize kernels by DIC treatment which increased the specific surface area. Moreover, as seen in Figure 6, under steam pressure values up to 0.4 MPa, the pericarp of some maize kernels was broken and it seems that this structural change allowed the water entry. As indicated by Ramos et al. [45], the entry of water into the kernels occurs predominantly through the pericarp, not through the tip cap.

On the other hand, by comparing both the controls and the DIC treatments, it can be observed that under 0.4 MPa and nine cycles of 10 s, it was possible to achieve in only 5 min the same moisture content of controls after 2 h of rehydration (0.4421 g H_2_O/g dry matter). Moreover, to obtain an average final moisture content of 0.7121 g H_2_O/g dry matter, DIC 17 and DIC 18 required 2 h instead of 17 h for controls. Similar results were found by Martínez-Garza [42] for blue, yellow, and white non-treated maize kernels which required 15 h of rehydration to achieve equilibrium moisture contents of 0.5270, 0.5000, and 0.5040 g H_2_O/g dry matter, respectively. This result reflects that DIC treatment intensified both rehydration stages, the starting accessibility and diffusional stages. Moreover, it has been stated that in this study rehydration kinetics were carried out at 20 °C, then, it can be suggested to study the rehydration kinetics of DIC-CAD samples under at least three different temperatures (i.e., 25, 35, and 45 °C) and on different varieties of maize kernels.

### 4.5. Water Holding Capacity

The water holding capacity (WHC) is a functional property of food that indicates how macro and micronutrients interact with water when force is applied. This property is fundamental in food formulations because it contributes significantly to food texture. In this study, complete maize flour was studied, and results showed that under the selected studied parameters of DIC, the WHC could be increased under high steam pressure and treatment time. As can be observed in Table 3, under DIC treatments of 0.4 MPa and 90 s, DIC allowed for increasing the WHC of maize flour to 1.6 (only cycle) and 2.4 times (nine cycles of 10 s) with respect to the controls. This increase in WHC generated by DIC treatment could be linked to a possible pre-gelatinization of maize starch at 0.4 MPa, equivalent to a steam pressure temperature of 143.6 °C. According to Phiarais and Arendt [46], to ensure the endosperm disruption and starch gelatinization of maize, it is necessary to exceed 100 °C. Thus, DIC as a thermomechanical treatment allowed a fast heating and instant cooling of the product. In this respect, studies need to be carried out to evaluate the impact of DIC treatment on the micro and macronutrients of maize kernels. Moreover, as indicated by Zondo and Mahlambi [47], the water holding capacity is also influenced by protein content and porosity attributes. Therefore, further studies are needed to evaluate the impact of DIC treatment on the micro and macronutrients of maize kernels.

### 4.6. Feasibility of Industrial Applications

It is worth noting that scaling up at the industrial level of maize drying intensification through DIC technology can absolutely be achievable. The DIC technology has been scaled up at the industrial level since 2001. The French company ABCAR DIC process (https://www.abcar-dic.com/en/, accessed on 26 June 2022) has developed DIC machines for fundamental research and industrial applications. Nowadays, industrial DIC equipment exists in various countries (e.g., Mexico, China, the USA, Canada, Italy,…) where the DIC technology is used not only for drying operations but also for microbial decontamination and bioactive compound extraction and allergen and non-nutritional compound reductions.

## 5. Conclusions

This study focused on the effect of the Instant Controlled Pressure Drop (DIC) technology on the drying and rehydration kinetics of maize kernels. Results showed that by comparing DIC treatments to CAD-only samples, DIC (0.4 MPa and 90 s) allowed for the reduction in the drying time from 180 min to ~105 min. However, under selected airflow conditions (0.4 m/s), it was impossible to guarantee negligible external resistances (NER) to dry processes; thereby, it was impossible to determine the effective diffusivity and the starting accessibility from experimental drying kinetics data. Further studies under different temperatures and several velocities of air flux, up to critical airflow velocity (CAV), will be needed to better evaluate the effect of DIC treatment on drying kinetics.

Moreover, DIC treatment intensified both rehydration stages: the starting accessibility stage and the diffusional stage. DIC (P = 0.4 MPa, t = 90 s divided into nine cycles of 10 s) achieved in only 3.5 min the same moisture content that controls had after 1 h of rehydration (0.40 g H_2_O/g dry matter). DIC also improved the WHC with respect to controls. DIC (0.4 MPa and nine cycles of 10 s) increased the WHC 2.3 times compared to the controls. Those results could suggest a microporous structural change in maize kernels explained by the autovaporization process during DIC treatment.

Finally, this preliminary study establishes the bases for developing an innovative maize postharvest drying operation by coupling the Instant Controlled Pressure Drop technology (DIC) to traditional Convective Air Drying (CAD).

## Figures and Tables

**Table 1:** Experimental Design Layout for the DIC treatments of maize kernels.

Assay No.	Run Order	Coded Values		Uncoded Values	
		Pressure (MPa)	Time (s)	Pressure (MPa)	Time (s)
1	DIC 2	−1	−1	0.14	22
2	DIC 11	1	−1	0.36	22
3	DIC 3	−1	1	0.14	78
4	DIC 12	1	1	0.36	78
5	DIC 1	–1.414	0	0.1	50
6	DIC 13	1.414	0	0.4	50
7	DIC 4	0	−1.414	0.25	10
8	DIC 10	0	1.414	0.25	90
9	DIC 9	0	0	0.25	50
10	DIC 5	0	0	0.25	50
11	DIC 6	0	0	0.25	50
12	DIC 8	0	0	0.25	50
13	DIC 7	0	0	0.25	50
14	DIC 14	1.414	1.414	0.4	90
15	DIC 15	1.414	1.414	0.4	90
16	DIC 16	1.414	1.414	0.4	90
17	DIC 17	1.414	1.414	0.4	10 s × 9 times
18	DIC 18	1.414	1.414	0.4	10 s × 9 times
19	DIC 19	1.414	1.414	0.4	10 s × 9 times
Control	NA	NA	NA	0	0

**Table 2:** Effect of DIC treatment on the thousand kernel weight and apparent drying coefficient of maize kernels.

Sample	P (MPa)	t (s)	Thousand Kernel Weight (g)	D_app_ (10^−10^ m²/s)
Control	NA	NA	272.02	0.3364
DIC 1	0.1	50	289.48	0.2904
DIC 2	0.14	22	311.77	0.3563
DIC 3	0.14	78	275.53	0.2237
DIC 4	0.25	10	265.67	0.2541
DIC 5	0.25	50	248.06	0.2041
DIC 6	0.25	50	276.21	0.2266
DIC 7	0.25	50	306.35	0.3175
DIC 8	0.25	50	277.82	0.1873
DIC 9	0.25	50	236.45	0.1738
DIC 10	0.25	90	289.67	0.2296
DIC 11	0.36	22	275.52	0.1928
DIC 12	0.36	78	305.52	0.2351
DIC 13	0.4	50	279.19	0.2017
DIC 14	0.4	90	287.63	0.3741
DIC 15	0.4	90	265.68	0.1982
DIC 16	0.4	90	276.83	0.2374
DIC 17	0.4	10 s × 9	279.38	0.2010
DIC 18	0.4	10 s × 9	241.64	0.2251
DIC 19	0.4	10 s × 9	260.05	0.3039

P = Steam pressure (MPa); t = Total treatment time (s); and D_app_ = Apparent drying coefficient (10^−10^ m²/s).

**Table 3:** Experimental data of the drying kinetics of maize kernels. The evolution of moisture content in dry basis (g H_2_O/g dry matter) in the time W = f(t) function.

Time (min)	Sample																					
	Control 1	Control 2	Control 3	DIC1	DIC2	DIC3	DIC4	DIC5	DIC6	DIC7	DIC8	DIC9	DIC 10	DIC 11	DIC 12	DIC 13	DIC 14	DIC 15	DIC 16	DIC 17	DIC 18	DIC 19
0	0.2553	0.2760	0.2506	0.2812	0.2559	0.2365	0.2483	0.2506	0.2425	0.2600	0.2692	0.2551	0.2624	0.2362	0.2312	0.2271	0.2406	0.2354	0.2517	0.2465	0.2523	0.2628
5	0.2383	0.2629	0.2379	0.2468	0.2261	0.2078	0.2197	0.2259	0.2217	0.2259	0.2397	0.2301	0.2330	0.2157	0.2067	0.2065	0.2199	0.2148	0.2435	0.2299	0.2357	0.2460
10	0.2256	0.2498	0.2295	0.2296	0.2090	0.1955	0.2074	0.2135	0.2093	0.2132	0.2271	0.2176	0.2246	0.2034	0.1944	0.1942	0.2035	0.2025	0.2229	0.2132	0.2191	0.2209
15	0.2128	0.2411	0.2169	0.2167	0.1963	0.1873	0.1951	0.1970	0.2010	0.2046	0.2187	0.2092	0.2120	0.1911	0.1903	0.1900	0.1952	0.1943	0.2105	0.2048	0.2067	0.1999
20	0.2085	0.2148	0.2127	0.2081	0.1877	0.1833	0.1910	0.1928	0.1927	0.1961	0.2103	0.2051	0.2037	0.1869	0.1821	0.1818	0.1870	0.1861	0.1982	0.1965	0.1984	0.1999
30	0.1915	0.2017	0.2000	0.1952	0.1792	0.1710	0.1787	0.1804	0.1802	0.1834	0.1935	0.1884	0.1911	0.1746	0.1699	0.1694	0.1746	0.1697	0.1858	0.1840	0.1860	0.1831
45	0.1745	0.1799	0.1790	0.1780	0.1622	0.1587	0.1624	0.1639	0.1677	0.1706	0.1767	0.1759	0.1743	0.1582	0.1576	0.1530	0.1622	0.1574	0.1693	0.1632	0.1694	0.1663
60	0.1660	0.1668	0.1664	0.1651	0.1537	0.1464	0.1501	0.1516	0.1594	0.1578	0.1641	0.1592	0.1617	0.1500	0.1453	0.1489	0.1540	0.1492	0.1570	0.1548	0.1569	0.1537
90	0.1490	0.1537	0.1537	0.1436	0.1367	0.1300	0.1337	0.1309	0.1386	0.1408	0.1431	0.1425	0.1408	0.1336	0.1289	0.1241	0.1334	0.1328	0.1405	0.1381	0.1445	0.1411
120	0.1362	0.1406	0.1411	0.1307	0.1196	0.1177	0.1214	0.1185	0.1262	0.1280	0.1263	0.1258	0.1282	0.1212	0.1167	0.1077	0.1128	0.1163	0.1199	0.1215	0.1320	0.1244
150	0.1277	0.1318	0.1285	0.1135	0.1111	0.1055	0.1092	0.1062	0.1095	0.1110	0.1137	0.1133	0.1114	0.1089	0.1085	0.1036	0.1087	0.1081	0.1158	0.1131	0.1196	0.1160
180	0.1192	0.1231	0.1200	0.1006	0.0983	0.0973	0.0969	0.0938	0.1012	0.0982	0.1011	0.1008	0.1030	0.1007	0.1003	0.0953	0.1004	0.0958	0.1035	0.1048	0.1113	0.1076

**Table 4:** Experimental data of the rehydration kinetics of maize kernels. Evolution of moisture content in dry basis (g H_2_O/g dry matter) as a function of the time W = f(t).

Time (min)	Sample																					
	Control 1	Control 2	Control 3	DIC 1	DIC 2	DIC 3	DIC 4	DIC 5	DIC 6	DIC 7	DIC 8	DIC 9	DIC 10	DIC 11	DIC 12	DIC 13	DIC 14	DIC 15	DIC 16	DIC 17	DIC 18	DIC 19
0	0.1192	0.1231	0.1200	0.1006	0.0983	0.0973	0.0969	0.0938	0.1012	0.0982	0.1011	0.1008	0.1030	0.1007	0.1003	0.0953	0.1004	0.0958	0.1035	0.1048	0.1113	0.1076
5	0.3078	0.3748	0.3113	0.3174	0.3128	0.2834	0.3108	0.2890	0.3452	0.3346	0.3455	0.3173	0.3337	0.3452	0.3183	0.3268	0.3794	0.3328	0.3397	0.4905	0.5150	0.3843
10	0.3106	0.3761	0.3200	0.3320	0.3436	0.3177	0.3374	0.3285	0.3606	0.3507	0.3505	0.3332	0.3576	0.3474	0.3329	0.3309	0.4176	0.3381	0.3547	0.5137	0.5794	0.4295
15	0.3286	0.3768	0.3550	0.3457	0.3514	0.3227	0.3528	0.3447	0.3716	0.3634	0.3744	0.3571	0.3578	0.3476	0.3463	0.3426	0.4210	0.3839	0.3559	0.5309	0.5917	0.4648
30	0.3441	0.3858	0.3606	0.3601	0.3589	0.3435	0.3716	0.3580	0.3722	0.3637	0.3880	0.3779	0.3795	0.3507	0.3590	0.4048	0.4551	0.3896	0.3911	0.5702	0.6006	0.5076
45	0.3607	0.4077	0.3643	0.3977	0.3656	0.3466	0.3818	0.3700	0.3945	0.3672	0.4194	0.3810	0.3881	0.4086	0.3707	0.4315	0.5205	0.4117	0.4077	0.6423	0.6425	0.5550
60	0.3971	0.4412	0.3666	0.3982	0.3855	0.3790	0.3885	0.3717	0.4130	0.3712	0.4323	0.3918	0.4119	0.4104	0.3848	0.4459	0.5448	0.4275	0.4684	0.6538	0.6866	0.5569
90	0.3974	0.4721	0.3995	0.4071	0.3973	0.3798	0.3985	0.3966	0.4467	0.3801	0.4428	0.4226	0.4300	0.4131	0.4173	0.4987	0.5592	0.4293	0.4847	0.6819	0.7050	0.6096
120	0.4279	0.4802	0.4181	0.4253	0.4344	0.3917	0.4158	0.4059	0.4532	0.4354	0.4681	0.4377	0.4379	0.4137	0.4239	0.5067	0.5632	0.4396	0.4860	0.7530	0.7434	0.6400
150	0.4504	0.4890	0.4742	0.4409	0.4443	0.4202	0.4257	0.4403	0.4825	0.4519	0.4814	0.4785	0.4542	0.4427	0.4755	0.5165	0.6083	0.4774	0.5320	0.7694	0.7509	0.6404
180	0.4784	0.5401	0.4868	0.4564	0.5147	0.4392	0.4795	0.4501	0.4917	0.4656	0.4988	0.4876	0.4649	0.4463	0.5075	0.5457	0.6305	0.5031	0.5519	0.8083	0.8406	0.6780

**Table 5:** Effect of DIC treatment on the rehydration kinetic parameters of maize kernels.

Sample	P	t	D_eff__-rehy_ (10^−10^ m²/s)	δW_s_	W_t = 120 min_	WHC
Control	NA	NA	0.0146	0.1975	0.4420	1.4304
DIC 1	0.1	50	0.0243	0.2146	0.4253	1.3531
DIC 2	0.14	22	0.0082	0.1961	0.4344	1.2859
DIC 3	0.14	78	0.0147	0.1737	0.3917	1.4913
DIC 4	0.25	10	0.0113	0.2001	0.4158	1.4822
DIC 5	0.25	50	0.0138	0.1695	0.4059	1.6066
DIC 6	0.25	50	0.0138	0.2406	0.4532	1.4571
DIC 7	0.25	50	0.0030	0.2235	0.4354	1.4515
DIC 8	0.25	50	0.0169	0.2475	0.4681	1.4524
DIC 9	0.25	50	0.0105	0.2036	0.4377	1.3685
DIC 10	0.25	90	0.0086	0.2257	0.4379	1.4828
DIC 11	0.36	22	0.0193	0.2589	0.4137	1.5135
DIC 12	0.36	78	0.0072	0.2123	0.4239	1.9006
DIC 13	0.4	50	0.0385	0.2436	0.5067	1.6806
DIC 14	0.4	90	0.0237	0.2809	0.5632	2.3838
DIC 15	0.4	90	0.0144	0.2309	0.4396	2.3359
DIC 16	0.4	90	0.0151	0.2486	0.4860	2.3620
DIC 17	0.4	10 s × 9	0.0250	0.3941	0.7530	3.2004
DIC 18	0.4	10 s × 9	0.0237	0.3756	0.7434	3.1834
DIC 19	0.4	10 s × 9	0.0315	0.2480	0.6400	3.5073

P = Steam pressure (MPa); t = Total treatment time (s); D_eff-rehy_ = Rehydration effective diffusivity (10^−10^ m²/s); δW_s_ = Starting Accessibility of Rehydration (g H_2_O/g dry matter); W_t = 120 min_ = Water content after 2 h of rehydration (g water/g dry matter); and WHC = water holding capacity (g of absorbed H_2_O/g dry sample).

## Data Availability

All related data and methods are presented in this paper. Additional inquiries should be addressed to the corresponding author.

## Nomenclature

*ρ_w_*	apparent density of water in the material (kg m^−3^)
*ρ_m_*	apparent density of solid material (kg m^−3^)
*v_w_*	absolute velocity of water flow within the porous medium (m s^−1^)
*v_m_*	absolute velocity of solid medium (m s^−1^)
*W*	water content (kg water/kg dry matter) in the solid matrix
*W*_1_	water content at the starting diffusion time
*W*_0_	value of moisture content calculated from diffusion model extrapolated to t = 0 (% db)
*W*_∞_	equilibrium water content at a very long time t → ∞ (kg water/kg dry matter)
*W_i_*	initial water content (kg water/kg dry matter)
*D_eff_*	effective diffusivity of water within the solid medium (m^2^ s^−1^) for dehydration or rehydration
D_app_	apparent drying coefficient (m^2^ s^−1^)
*d_p_*	thickness (m)
*k*	slope of y = Ln (Moisture Ratio) as a function of time (s^−1^)
δW_s_	starting accessibility of water (kg water/kg dry matter) for dehydration d or rehydration r
*τ*	Fick’s number
*A_i_*, *q_i_*	Crank’s coefficients according to the geometry of solid matrix
